# Long noncoding RNA Kcnq1ot1 prompts lipopolysaccharide-induced acute lung injury by microRNA-7a-5p/Rtn3 axis

**DOI:** 10.1186/s40001-022-00653-8

**Published:** 2022-03-22

**Authors:** Shuo Yang, Fang Liu, Di Wang

**Affiliations:** 1 Department of Geriatrics, Daqing Qilfield General Hospital, Daqing, 163000 Heilongjiang China; 2 Department of Prosthodontics, Daqing Qilfield General Hospital, Zhongkang Street No. 9, Sartu District, Daqing, 163000 Heilongjiang China

**Keywords:** Acute lung injury, Lipopolysaccharide, Long noncoding RNA KCNQ1 overlapping transcript 1, MicroRNA-7a-5p, Reticulon 3, Inflammation, Apoptosis

## Abstract

**Background:**

Long noncoding RNA (lncRNA)-regulated mechanism in acute lung injury (ALI) has attracted special interests in study researches. We planned to disclose whether KCNQ1 overlapping transcript 1 (Kcnq1ot1) is involved in ALI and its mechanism.

**Methods:**

The lipopolysaccharide (LPS)-induced ALI model was established in mice. Kcnq1ot1, microRNA (miR)-7a-5p and Reticulon 3 (Rtn3) levels were measured in lung tissues of mice. The vector that changed Kcnq1ot1, miR-7a-5p and Rtn3 expression was injected into LPS-treated mice, and pathological damage, fibrosis, apoptosis and inflammatory response were subsequently examined in lung tissues. The relation between Kcnq1ot1 and miR-7a-5p, and that between miR-7a-5p and Rtn3 were identified.

**Results:**

Kcnq1ot1 and Rtn3 expression increased while miR-7a-5p expression decreased in LPS-treated mice. Reduced Kcnq1ot1 or elevated miR-7a-5p alleviated pathological damage, fibrosis, apoptosis and inflammatory response in ALI mice, while overexpressed Rtn3 worsened ALI in mice. Downregulation of Rtn3 reversed the exacerbation of miR-7a-5p downregulation in ALI mice. Kcnq1ot1 competitively bound to miR-7a-5p and miR-7a-5p negatively mediated Rtn3 expression.

**Conclusion:**

Our experiments evidence that silencing Kcnq1ot1 upregulates miR-7a-5p to suppress Rtn3 expression, thereby diminishing LPS-induced ALI.

**Supplementary Information:**

The online version contains supplementary material available at 10.1186/s40001-022-00653-8.

## Introduction

Acute lung injury (ALI) is a moderate or mild form of acute respiratory distress syndrome (ARDS) [1]. Clinically, the criteria for ALI manifests as progressive hypoxemia, dyspnea and increased work of breathing [2]. ALI is characterized by the recruitment of neutrophils to the lung, accompanied by alveolar and systemic release of chemokines, pro-inflammatory cytokines, acute phase reactants, and matrix remodeling enzymes [3]. The need for positive end-expiratory pressure and high FIO2 mechanical ventilation is a treatment guideline for patients with ALI [4]. The current drugs include surfactants and neuromuscular blockers to limit medical treatment, vasodilators and anticoagulants to optimize alveolar perfusion and diuretics and β2 agonists to reduce pulmonary edema [5]. Although with pharmacological drugs and modern technique care, it is still essential to translate the mechanism of ALI for further exploration of treatment therapeutics.

Long noncoding RNAs (lncRNAs) refer to a family of transcripts that involve in biological processes ranging from housekeeping functions to more specialized functions [6]. A large fraction of lucernes have been implicated in ALI and lncRNAs-based therapy is of importance to control the disease progression. For instance, overexpressing lncRNA cancer susceptibility candidate 2 could improve ALI through suppression of lung epithelial cell apoptosis [7]. Suppression of lncRNA metastasis associated in lung adenocarcinoma transcript 1 [8] or lncRNA maternally expressed gene 3 restrains inflammatory response [9] in ALI. KCNQ1 overlapping transcript 1 (Kcnq1ot1) is a member of lncRNA family that confers oncogenesis in the lung [10]. In lipopolysaccharide (LPS)-induced ARDS, Kcnq1ot1 silencing is of significance in restraining inflammatory insult [11]. LncRNA often exerts functions through acting as a sponge of microRNA (miR). miR-7 protects against pulmonary fibrosis by decreasing epithelial–mesenchymal transition of bronchial epithelial cells [12]. The protective role of miR-7a-5p has been shown in LPS-induced myocardial injury [13] as well as in LPS-mediated microglial inflammation [14]. Reticulon (Rnt) protein family has been implicated in the modification of tissue repair and acute inflammation. For example, Nogo-B could bat against LPS-induced ALI [15]. Of a member of Rnt, Rtn3 was found to have a potential targeting relation with miR-7a-5p online. Definitely, Fu et al. have profiled the pro-inflammatory property of Rtn3 in osteoarthritis [16]. Till now, little few relevant documents have discovered the relative mechanism underlying ALI with attention on Kcnq1ot1-mediated miR-7a-5p/Rtn3 axis. Given that, we started the research to unravel the mechanistic program of Kcnq1ot1/miR-7a-5p/Rtn3 feedback loop in LPS-induced ALI.

## Materials and methods

### Ethics statement

The animal research was approved by the Ethics Committee of Daqing Qilfield General Hospital.

### Animals

Six-week-old male BALB/c mice (18–22 g) were kept in a specific pathogen-free laboratory (22–26 °C, 40–70% humidity) with day/night cycle. The mice were reared in different cages (4 mice per cage) and fed and drank freely for a week [17].

### Modeling of animals

The mice were given intratracheal instillation of LPS at 3 mg/kg. The method of LPS instillation was applied to establish ALI model. At 6 h post LPS instillation, mice were injected intraperitoneally with sh-negative control (NC), sh-Kcnq1ot1, agomiR-NC, miR-7a-5p agomiR, pcDNA-NC, pcDNA-Rtn3, miR-7a-5p antagomiR + sh-NC, and miR-7a-5p antagomiR + sh-Rtn3, respectively. The injection dose was 10 μmol/kg. At 48 h post the injection with vectors, the mice were euthanized 6 h later to obtain lung tissue specimens [18]. All vectors were provided by GenePharma (Shanghai, China).

### Hematoxylin–eosin (H&E) staining

The lung tissue was dehydrated by ethanol gradient and embedded in paraffin after permeabilization with xylene. The sectsions (4 μm) were conventionally dehydrated, stained with hematoxylin, differentiated with 1% alcohol hydrochloride and dyed with eosin. Afterwards, gradient alcohol was added in the section for dehydration and xylene for permeabilization. Finally, neutral resin-sealed sections were observed under an optical microscope [19].

### Masson staining

The tissue was stained with red algae fuchsin solution, rinsed with glacial acetic acid, immersed in phosphomolybdic acid, and dyed with aniline blue solution. After that, the sample was soaked in glacial acetic acid, treated with xylene and observed by an electron microscope [20].

### Transferase-mediated deoxyuridine triphosphate-biotin nick end labeling (TUNEL) staining

The lung tissue was treated with in situ cell death detection kit (Roche, Switzerland) and observed in 5 fields of view to calculate the rate of TUNEL-positive cells [21].

### Reverse transcription quantitative polymerase chain reaction (RT-qPCR)

The lung tissue was processed with Trizol (Invitrogen, CA, USA) to extract total RNA which was reverse-transcribed into cDNA by PrimeScript™ RT kit (Thermo Fisher Scientific, MA, USA) or All-in-One miRNA’s first chain CDNA Synthesis Kit (GeneCopoeia, MD, USA). cDNA was then treated with Lightcycler 480 96-well PCR (Roche, Mannheim, Germany). The internal controls to standardize gene expression were glyceraldehyde-3-phosphate dehydrogenase (GAPDH) and U6. Additional file 3: Table S1 lists the primer sequences. The gene calculation method was 2^−ΔΔCt^ method [22].

### Western blot assay

The lung tissue was lysed by radio-immunoprecipitation assay buffer (Thermo Fisher Scientific) supplemented with protease inhibitors, after which protein level was evaluated by a bicinchoninic acid protein detection kit (Thermo Fisher Scientific). The protein was analyzed with sodium dodecyl sulfate polyacrylamide gel electrophoresis. Subsequently, the protein sample on the polyvinylidene fluoride membrane was blocked, combined with primary antibodies Rtn3 and GAPDH (both from Santa Cruz Biotechnology, CA, USA), and with peroxidase-labeled secondary antibody (Abcam, CA, USA). The protein bands were analyzed with Odyssey 3.0 [23].

### Dual luciferase reporter gene assay

The synthesized wild-type (WT) or mutant-type (MUT) Kcnq1ot1 and Rtn3 3′UTR sequences (Generalbio, Anhui, China) containing miR-7a-5p binding site was utilized to form Kcnq1ot1-WT, Rtn3-WT, Kcnq1ot1-MUT and Rtn3-MUT reporters. By Lipofectamine 3000 (Invitrogen), the reporter was co-transfected with miR-7a-5p mimic or miR-7a-5p NC into HEK293T cells to assess cell luciferase activity with dual luciferase reporter gene test kit (Beyotime, Shanghai, China) [24].

### RNA immunoprecipitation (RIP) assay

 Cells were lysed in RIP lysis buffer (protease inhibitors and RNase inhibitors). Protein-G/A + agarose and Ago2 (Abcam) or rabbit IgG (Abcam) were incubated overnight, the precipitated RNA was subjected to RT-qPCR [25].

### Statistical analysis

SPSS 21.0 (IBM, NY, USA) and GraphPad Prism 6 Software (San Diego, CA, USA) were the data analysis software. Measurement data were expressed as mean ± standard deviation. Two groups of measurement data were compared by *t* test, while multiple groups of data were analyzed by analysis of variance and Tukey’s post hoc test. *P* < 0.05 represented statistical significance.

## Results

### Kcnq1ot1 and Rtn3 expression increases while miR-7a-5p expression decreases in mice with ALI

**Fig. 1:**
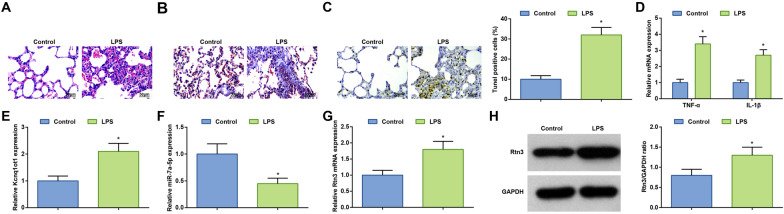
Kcnq1ot1 and Rtn3 expression increases while miR-7a-5p expression decreases in mice with ALI. **A** H&E staining; **B** Masson staining; **C** TUNEL staining; **D** TNF-α and IL-1β levels in LPS-treated mice; **E** Kcnq1ot1 mRNA expression in LPS-treated mice; **F** miR-7a-5p expression in LPS-treated mice; **G**, **H** Rtn3 protein expression in LPS-treated mice; measurement data are displayed as mean ± standard deviation (*n* = 6); * *P* < 0.05 vs. the Control group

Observation of lung tissue samples by HE staining, Masson staining and TUNEL staining depicted that in LPS-conditioned mice, the alveolar structure was disordered, the alveolar walls were thickened, and a large number of inflammatory cells and erythrocytes were seen in the alveolar cavity (Fig. 1A); diffuse fibrosis and severe damage were manifested (Fig. 1B); more apoptotic cells and brown particles were obviously detected (Fig. 1C). Measurements by ELISA revealed that TNF-α and IL-1β levels were promoted in mice after LPS treatment (Fig. 1D).

Kcnq1ot1, miR-7a-5p, and Rtn3 levels in lung tissues of mice were tested by RT-qPCR and Western blot. After LPS instillation, Kcnq1ot1 and Rtn3 levels went to upregulate and miR-7a-5p expression dropped in mice (Fig. 1E–H).

### Reduced Kcnq1ot1 alleviates the pathological degree of ALI in mice

**Fig. 2:**
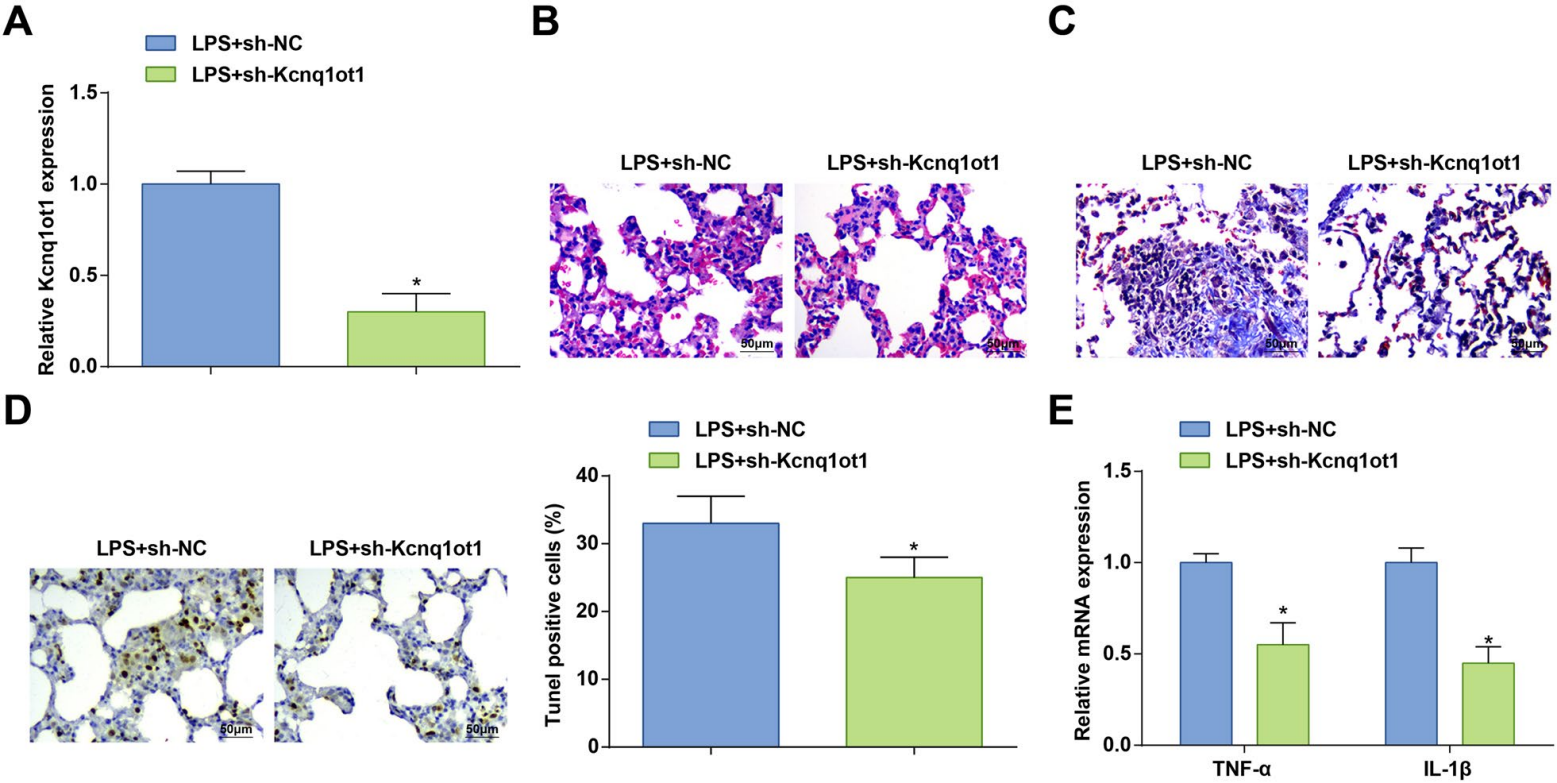
Reduced Kcnq1ot1 alleviates the pathological degree of ALI in mice. **A** Kcnq1ot1 expression in LPS-treated mice after injection with sh-Kcnq1ot1; **B** H&E staining; **C** Masson staining; **D** TUNEL staining; **E** TNF-α and IL-1β levels in LPS-treated mice after injection with sh-Kcnq1ot1; measurement data are displayed as mean ± standard deviation (*n* = 6); * *P* < 0.05 vs. the LPS + sh-NC group

Kcnq1ot1 is a key regulatory factor to prevent myocardial injury [26]. However, it is not clear about the mechanism of Kcnq1ot1 in LPS-induced ALI. LPS-treated mice were injected with sh-Kcnq1ot1 or sh-NC. RT-qPCR confirmed the efficacy of sh-Kcnq1ot1 to lower Kcnq1ot1 expression in the lung tissue (Fig. 2A). In response to Kcnq1ot1 silencing, the structure of the alveoli was improved, the alveolar wall no longer thickened, and the number of inflammatory cells and erythrocytes were reduced (Fig. 2B), the diffuse fibrosis and damage of the lung tissue were improved (Fig. 2C), the apoptosis rate was reduced (Fig. 2D), and TNF-α and IL-1β levels were ablated (Fig. 2E) in LPS-treated mice. In summary, silent Kcnq1ot1 reduces the degree of LPS-induced ALI.

### Kcnq1ot1 competitively binds to miR-7a-5p

On the bioinformatics website DIANA, the presence of binding site between Kcnq1ot1 and miR-7a-5p was confirmed (Additional file 1: Fig. S1A). In the dual luciferase gene reporter detection, miR-7a-5p mimic caused luciferase activity inhibited in the Kcnq1ot1-WT reporter (Additional file 1: Fig. S1B). In RIP analysis, Kcnq1ot1 level Ago was increased by miR-7a-5p mimic (Additional file 1: Fig. S1C).

Besides, the finding of RT-qPCR exhibited that silencing Kcnq1ot1 increased the level of miR-7a-5p in the lung tissue of LPS-treated mice (Additional file 1: Fig. S1D). Therefore, Kcnq1ot1 worked as a molecular sponge for miR-7a-5p.

### Elevated miR-7a-5p reduces ALI in mice

**Fig. 3:**
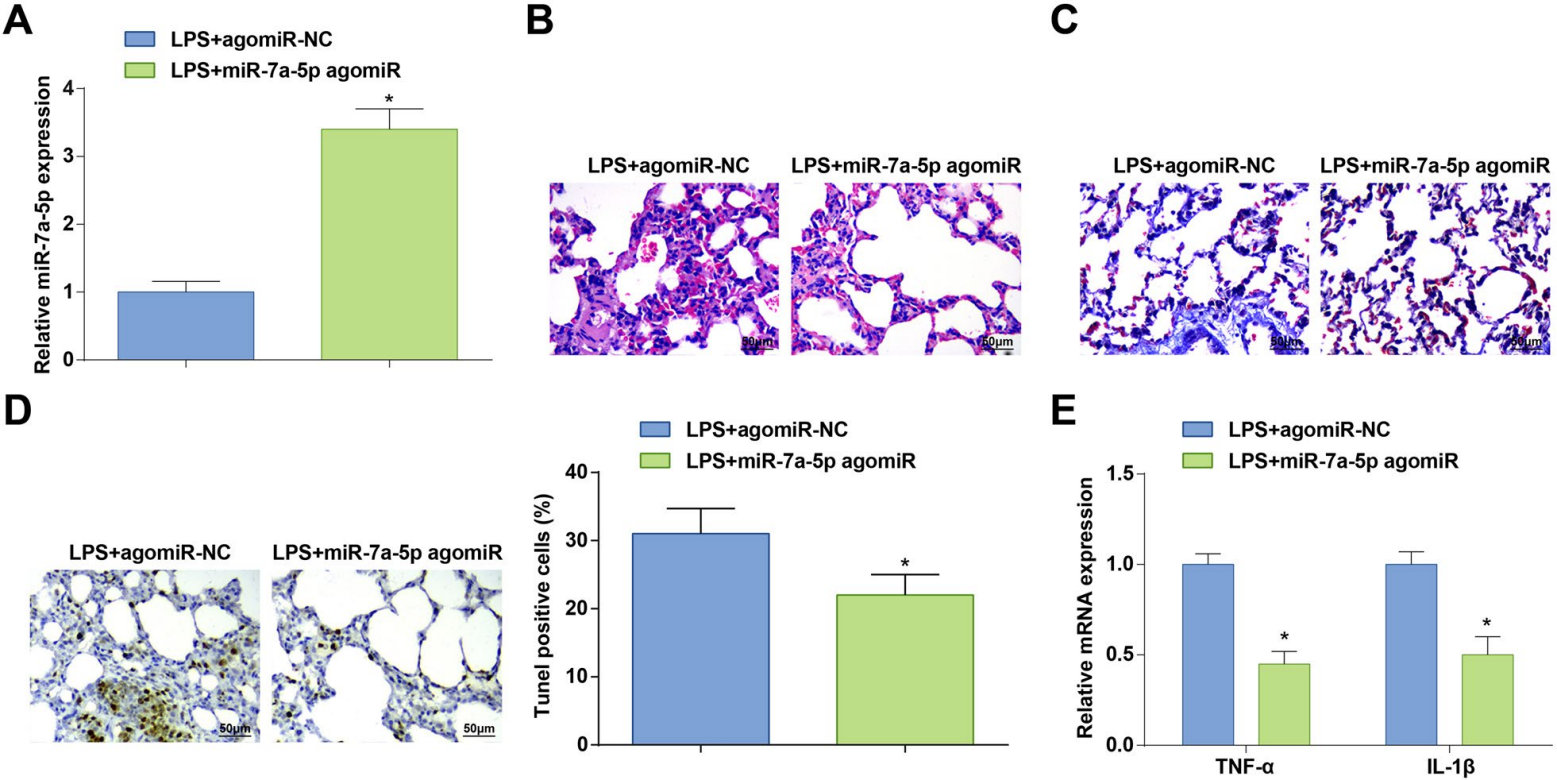
Elevated miR-7a-5p reduces ALI in mice. **A** miR-7a-5p expression in LPS-treated mice after injection with miR-7a-5p agomiR; **B** H&E staining; **C** Masson staining; **D** TUNEL staining; **E** TNF-α and IL-1β levels in LPS-treated mice after injection with miR-7a-5p agomiR; measurement data are displayed as mean ± standard deviation (*n* = 6); * *P* < 0.05 vs. the LPS + agomiR-NC group

LPS-treated mice were injected with miR-7a-5p agomiR or miR-7a-5p NC. RT-qPCR detected that miR-7a-5p agomiR successfully raised miR-7a-5p expression in ALI mice (Fig. 3A). After miR-7a-5p overexpression, the pathological conditions were improved (Fig. 3B), the degree of lung fibrosis (Fig. 3C), the number of apoptotic cells and the level of inflammatory indices presented a decrease (Fig. 3D, E). It was indicated that overexpression of miR-7a-5p can improve ALI in mice.

### miR-7a-5p negatively mediates Rtn3 expression

On the starBase, the binding site of miR-7a-5p and Rtn3 was predicted (Additional file 2: Fig. S2A). In the dual luciferase gene reporter test, Rtn3-WT luciferase activity could be suppressed by miR-7a-5p mimic (Additional file 2: Fig. S2B). In the RIP experiment, the enrichment of Rtn3 was induced by miR-7a-5p mimic (Additional file 2: Fig. S2C). Additionally, in RT-qPCR and Western blot, Rtn3 expression was decreased in mice after upregulating miR-7a-5p (Additional file 2: Fig. S2D, E). Distinctly, miR-7a-5p and Rtn3 have a negative targeting relation.

### Overexpressed Rtn3 worsens ALI in mice

**Fig. 4:**
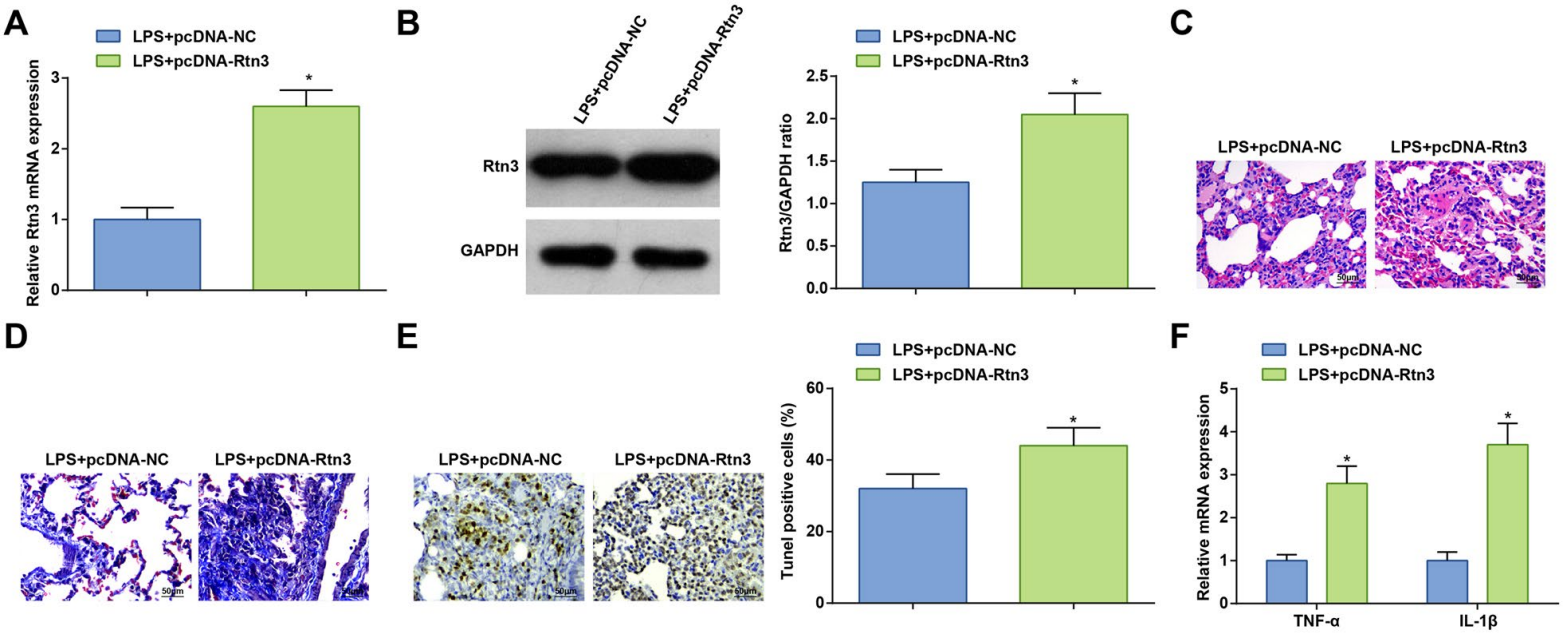
Overexpressed Rtn3 worsens ALI in mice. **A**, **B**. Rtn3 mRNA and protein expression in LPS-treated mice after injection with pcDNA-Rtn3; **C** H&E staining; **D** Masson staining; **E** TUNEL staining; **F** TNF-α and IL-1β levels in LPS-treated mice after injection with pcDNA-Rtn3; measurement data are displayed as mean ± standard deviation (*n* = 6); * *P* < 0.05 vs. the LPS + pcDNA-NC group

LPS-suffered mice were treated with pcDNA-Rtn3 or pcDNA-NC. RT-qPCR and Western blot identified that Rtn3 expression was substantially elevated by pcDNA-Rtn3 (Fig. 4A, B). As a result of Rtn3 overexpression, LPS-suffered mice presented deteriorated pathological condition of the lung tissue, more serious lung tissue fibrosis (Fig. 4C, D), increased number of apoptotic cells (Fig. 4E) and aggravated inflammatory response (Fig. 4F). Markedly, overexpressed Rtn3 worsens ALI in mice.

### miR-7a-5p suppresses Rtn3 expression to alleviate ALI

**Fig. 5:**
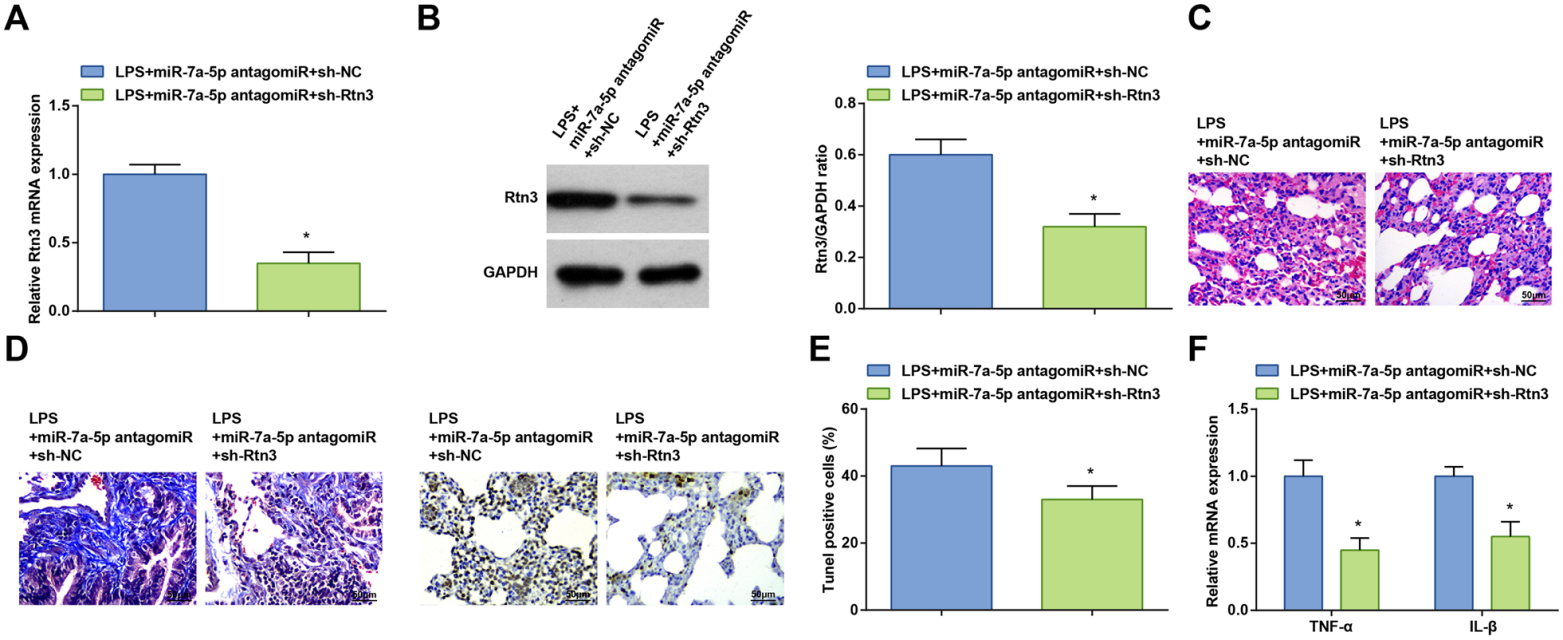
miR-7a-5p suppresses Rtn3 expression to alleviate ALI. **A**, **B** Rtn3 mRNA and protein expression in LPS-treated mice after injection with miR-7a-5p antagomiR and sh-Rtn3; **C** H&E staining; **D** Masson staining; **E** TUNEL staining; **F** TNF-α and IL-1β levels in LPS-treated mice; measurement data are displayed as mean ± standard deviation (*n* = 6); * *P* < 0.05 vs. the LPS + miR-7a-5p antagomiR + sh-NC group

Rescue tests were performed in LPS-treated mice. On the basis miR-7a-5p antagomiR, sh-Rtn3 was injected into mice to lower Rtn3 expression (Fig. 5A, B). Moreover, downregulating Rtn3 weakened the negative role of inhibited miR-7a-5p in ALI mice (Fig. 5D–F). Plainly, Kcnq1ot1 worsens ALI in mice by suppressing miR-7a-5p to promote Rtn3 expression.

## Discussion

ALI represents a serious heterogenous pulmonary disorder with high mortality. In experimental trails, direct intratracheal LPS instillation is a commonly used method to induce ALI in mice [27]. In mice with ALI induced by LPS, the manifestation and functions of Kcnq1ot1/miR-7a-5p/Rtn3 axis were partly explored. The main outcome elaborated that silencing Kcnq1ot1 made a relief of ALI in mice by upregulating miR-7a-5p to downregulate Rtn3.

To start with, Kcnq1ot1 expression was calculated to increase in the lung tissue of mice with ALI. Then, targeted silencing Kcnq1ot1 was performed to minimize injury in the lung of mice with ALI by alleviating alveolar injury and fibrosis, and reducing the secretion of inflammatory indices (TNF-α and IL-1β) and cell apoptosis. Exactly, knocking down Kcnq1ot1 in mice with ARDS is prerequisite to reduce inflammatory response through decreasing the level of pro-inflammatory actors and increasing anti-inflammatory parameter in ARDS induced by LPS [11]. It is of interest that Kcnq1ot1 expression reaches to a high level in neurons after stimulation with LPS, while repression of Kcnq1ot1 blocks apoptosis and inflammation to facilitate the survival of neurons [28]. High Kcnq1ot1 expression has been once measured in oxygen–glucose deprivation and reperfusion-conditioned neurons, but artificially restraining Kcnq1ot1 expression subsides inflammation and neuronal apoptosis [29]. Remarkably, cardiomyocytes subjected to hypoxic injury have high Kcnq1ot1 expression which notoriously foments cardiomyocyte injury and apoptosis [30]. Yang et al. have checked that loss of Kcnq1ot1 in mice with diabetic cardiomyopathy could rescue cardiac function and relieve fibrosis and pyroptosis [31]. An increase has been detected in Kcnq1ot1 expression in clinical cartilage tissues of patients with osteoarthritis, and depletion of Kcnq1ot1 is fundamental for obstructing the release of inflammatory cytokines and the degradation of extracellular matrix [32]. Generally, it is therapeutically potential to manage disease progression by taking control of the aberrant expression of Kcnq1ot1.

Subsequently, miR-7a-5p expression was inspected to downregulate in the lung tissue of mice with ALI. In fact, miR-7a-5p expression is limited to a relative low level in LPS-suffered cardiomyocytes [13]. Based on the binding relation with Kcnq1ot1, miR-7a-5p was speculated to involve in Kcnq1ot1-mediated ALI. Actually, the experimental outcomes displayed that restoring miR-7a-5p alone defensed against ALI in mice, but inhibiting miR-7a-5p lessened Kcnq1ot1 silencing-eased ALI. Du et al. have profiled that miR-7a-5p is downregulated in the case of diabetic kidney disease, but experimentally elevating miR-7a-5p expression in mice could prevent renal fibrosis to progress in the disease [33]. In a similar fashion, miR-7a-2-3p expression is prone to downregulate in oxygen–glucose deprivation-treated neurons, but the survival of cortical neurons is appreciative of the induction of miR-7a-2-3p [34]. Meng et al. have recognized an interesting fact that overexpressed miR-7a-5p detains the activation and inflammatory reaction of microglial [14]. To the best of our knowledge, inducing miR-7a/b in the heart of mice with myocardial infarction is the cornerstone of fibrosis and apoptosis inhibition [35].

The miRNA–mRNA network was also observed between miR-7a-5p and Rtn3 in ALI. Regarding the results, Rtn3 was upregulated in the lung of mice with ALI. Manipulated overexpression of Rtn3 deteriorated ALI in mice independently, whereas downregulating Rtn3 reduced the effect of miR-7a-5p silencing on mice with ALI. Regarding the biological functions of Rtn3, a study report on osteoarthritis has offered evidence that Rtn3 augments apoptosis and inflammatory response of chondrocytes [16]. Besides, in the setting of ischemia/reperfusion injury, the abnormal elevation of Rtn3 expression is an active stimuli for cardiomyocyte apoptosis [36].

## Conclusion

Overall, the present research proves that Kcnq1ot1 and Rtn3 are overexpressed, whereas miR-7a-5p is under-expressed in LPS-induced ALI. Suppression of Kcnq1ot1 elevates miR-7a-5p and reduces Rtn3 expression, so as to induce protection against ALI. The main finding in the present research provides a theoretical basis, promisingly carrying forward the development of molecule-targeted therapy for ALI. Meaningfully, evaluation of Kcnq1ot1/miR-7a-5p/Rtn3 axis discloses a novel direction for carrying forward treatments in ALI.

## Supplementary Information

**Additional file 1: Figure S1.** Kcnq1ot1 competitively binds to miR-7a-5p. **A** The binding sites of Kcnq1ot1 and miR-7a-5p on DIANA; **B**–**C** Targeting relationship between Kcnq1ot1 and miR-7a-5p verified by dual luciferase detection and RIP; **D** miR-7a-5p expression in LPS-treated mice after injection with sh-Kcnq1ot1; measurement data were displayed as mean ± standard deviation (*n* = 6); repetitions = 3; * *P* < 0.05 vs. the LPS + sh-NC group.

**Additional file 2: Figure S2.** miR-7a-5p negatively mediates Rtn3 expression. **A** The binding sites of miR-7a-5p and Rtn3 on StarBase; **B**–**C** Targeting relationship between miR-7a-5p and Rtn3 verified by dual luciferase detection and RIP; **D**–**E** Rtn3 mRNA and protein expression in LPS-treated mice after injection with miR-7a-5p agomiR; measurement data were displayed as mean ± standard deviation (*n* = 6); repetitions = 3; * *P* < 0.05 vs. the LPS + agomiR-NC group.

**Additional file 3: Table S1.** Primer sequences for genes in our article.

## Data Availability

Not applicable.

## Abbreviations

ALI: Acute lung injury
ARDS: Acute respiratory distress syndrome
lncRNAs: Long noncoding RNAs
Kcnq1ot1: KCNQ1 overlapping transcript 1
LPS: Lipopolysaccharide
miR: MicroRNA
Rnt: Reticulon
NC: Negative control
H&E: Hematoxylin–eosin
TUNEL: Transferase-mediated deoxyuridine triphosphate-biotin nick end labeling
ELISA: Enzyme-linked immunosorbent assay
TNF-α: Tumor necrosis factor-α
IL-1β: Interleukin-1β
RT-qPCR: Reverse transcription quantitative polymerase chain reaction
GAPDH: Glyceraldehyde-3-phosphate dehydrogenase
WT: Wild type
MUT: Mutant type
RIP: RNA immunoprecipitation
