# Supplementary figures and images for: Long noncoding RNA Kcnq1ot1 prompts lipopolysaccharide-induced acute lung injury by microRNA-7a-5p/Rtn3 axis
Source: Eur J Med Res. 2022 Mar 22;27:46. doi: 10.1186/s40001-022-00653-8 (PMC8939215; doi:10.1186/s40001-022-00653-8)

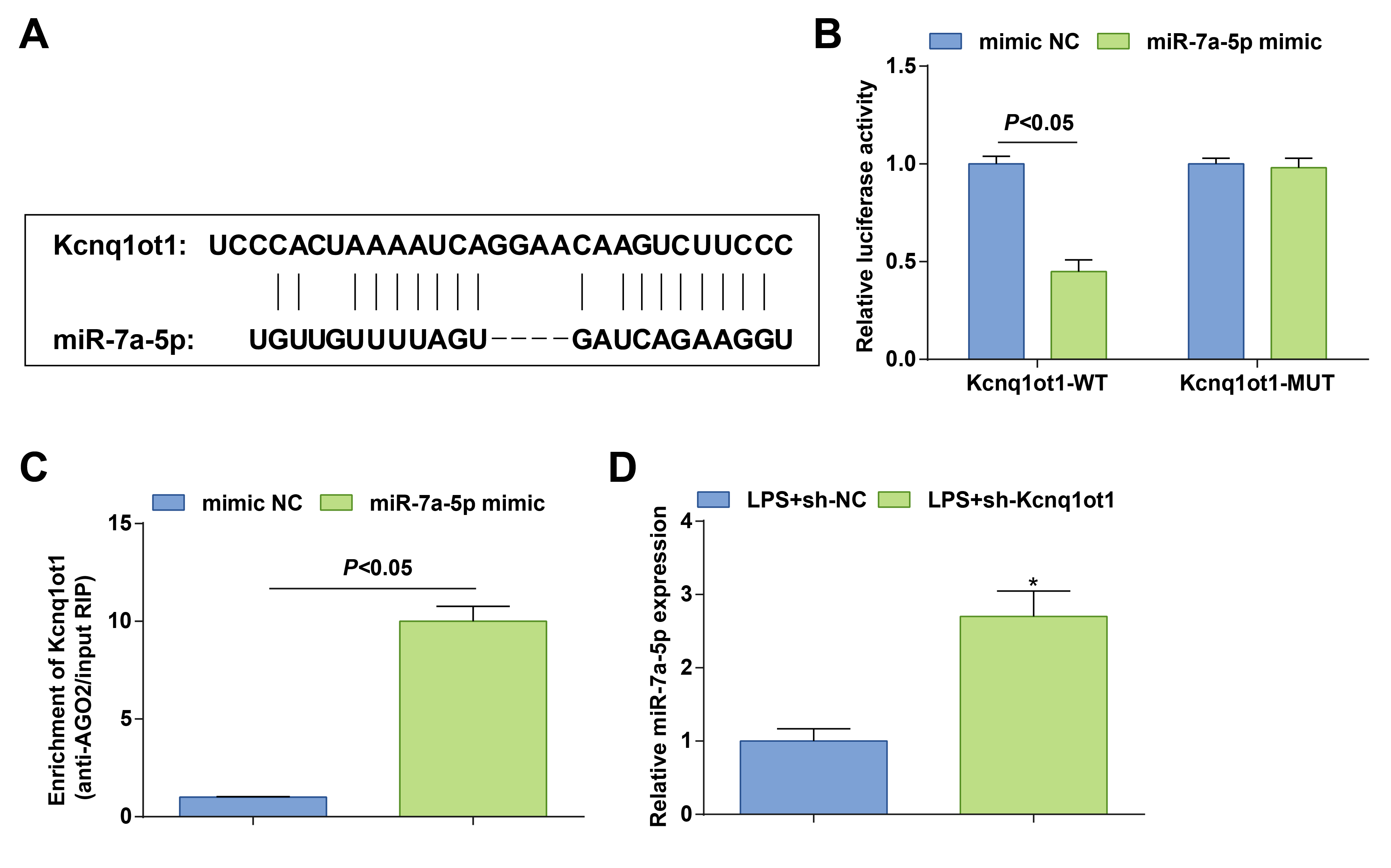

Supplement: Supplementary file 1 — Additional file 1: Figure S1. Kcnq1ot1 competitively binds to miR-7a-5p. A The binding sites of Kcnq1ot1 and miR-7a-5p on DIANA; B–C Targeting relationship between Kcnq1ot1 and miR-7a-5p verified by dual luciferase detection and RIP; D miR-7a-5p expression in LPS-treated mice after injection with sh-Kcnq1ot1; measurement data were displayed as mean ± standard deviation (n = 6); repetitions = 3; * P < 0.05 vs. the LPS + sh-NC group. [file 40001_2022_653_MOESM1_ESM.jpg]

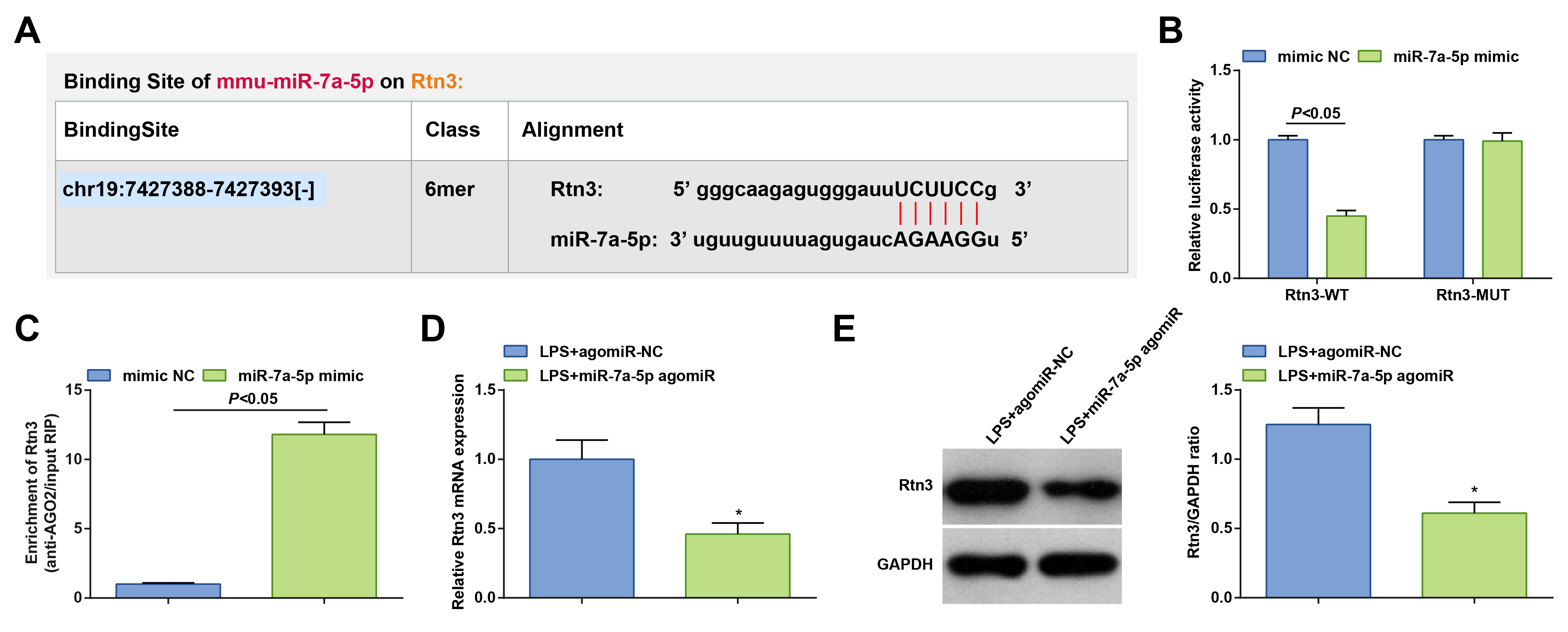

Supplement: Supplementary file 2 — Additional file 2: Figure S2. miR-7a-5p negatively mediates Rtn3 expression. A The binding sites of miR-7a-5p and Rtn3 on StarBase; B–C Targeting relationship between miR-7a-5p and Rtn3 verified by dual luciferase detection and RIP; D–E Rtn3 mRNA and protein expression in LPS-treated mice after injection with miR-7a-5p agomiR; measurement data were displayed as mean ± standard deviation (n = 6); repetitions = 3; * P < 0.05 vs. the LPS + agomiR-NC group. [file 40001_2022_653_MOESM2_ESM.jpg]
