# Supplementary material for: Long noncoding RNA Kcnq1ot1 prompts lipopolysaccharide-induced acute lung injury by microRNA-7a-5p/Rtn3 axis
Source: Eur J Med Res. 2022 Mar 22;27:46. doi: 10.1186/s40001-022-00653-8 (PMC8939215; doi:10.1186/s40001-022-00653-8)
Supplement: Supplementary file 3 — Additional file 3: Table S1. Primer sequences for genes in our article. [file 40001_2022_653_MOESM3_ESM.docx]

**Supplementary Table 1** Primer sequences for genes in our article

| Primers | Forward (5’→3’) | Reverse (5’→3’) |
| --- | --- | --- |
| Kcnq1ot1 | TGTCCCTTCTCACTGGAGCTG | GGTTACTCACACGGTGAAGTGG |
| miR-7a-5p | TGGAAGACTAGTGATTTTGTTGT | Universal primer |
| Rtn3 | AGGTGCCCCTACGATGTCTC | GGTTTGCTTGAGTTTTCCTCCA |
| TNF-α | ACCCTCACACTCAGATCATCT | TTGTCTTTGAGATCCATGCCGT |
| IL-1β | CACCTCACAAGCAGAGCACAAG | GCATTAGAAACAGTCCAGCCCATAC |
| U6 | GCTTCGGCAGCACATATACTAAAAT | CGCTTCACGAATTTGCGTGTCAT |
| GAPDH | CTGGAGAAACCTGCCAAGTATG | GGTGGAAGAATGGGAGTTGCT |

Note: Kcnq1ot1, KCNQ1 overlapping transcript 1, miR-7a-5p, microRNA-7a-5p; Reticulon 3, Rtn3; GAPDH, glyceraldehyde-3-phosphate dehydrogenase.
